# Development and evaluation of a spiral model of assessing EBM competency using OSCEs in undergraduate medical education

**DOI:** 10.1186/s12909-021-02650-7

**Published:** 2021-04-10

**Authors:** B. Kumaravel, C. Stewart, D. Ilic

**Affiliations:** 1 The University of Buckingham Medical School, Hunter Street, Buckingham, MK18 1EG UK; 2 University of Nottingham, Nottingham, UK; 3 School of Public Health and Preventive Medicine, Monash University, Melbourne, Australia

**Keywords:** Evidence-based medicine, Undergraduate medical curriculum, Competency, OSCEs, Summative

## Abstract

**Background:**

Medical students often struggle to understand the relevance of Evidence Based Medicine (EBM) to their clinical practice, yet it is a competence that all students must develop prior to graduation. Objective structured clinical examinations (OSCEs) are a valued assessment tool to assess critical components of EBM competency, particularly different levels of mastery as they progress through the course. This study developed and evaluated EBM based OSCE stations with an aim to establish a spiral approach for EBM OSCE stations for undergraduate medical students.

**Methods:**

OSCE stations were developed with increasingly complex EBM tasks. OSCE stations were classified according to the classification rubric for EBP assessment tools (CREATE) framework and mapped against the recently published core competencies for evidence-based practice (EBP). Performance data evaluation was undertaken using Classical Test Theory analysing mean scores, pass rates, and station item total correlation (ITC) using SPSS.

**Results:**

Six EBM based OSCE stations assessing various stages of EBM were created for use in high stakes summative OSCEs for different year groups across the undergraduate medical degree. All OSCE stations, except for one, had excellent correlation coefficients and hence a high reliability, ranging from 0.21–0.49. The domain mean score ranged from 13.33 to 16.83 out of 20. High reliability was demonstrated for the each of the summative OSCE circuits (Cronbach’s alpha = 0.67–0.85).

In the CREATE framework these stations assessed knowledge, skills, and behaviour of medical students in asking, searching, appraising, and integrating evidence in practice. The OSCE stations were useful in assessing six core evidence-based practice competencies, which are meant to be practiced with exercises. A spiral model of OSCEs of increasing complexity was proposed to assess EBM competency as students progressed through the MBChB course.

**Conclusions:**

The use of the OSCEs is a feasible method of authentically assessing leaner EBM performance and behaviour in a high stakes assessment setting. Use of valid and reliable EBM-based OSCE stations provide evidence for continued development of a hierarchy of assessing scaffolded learning and mastery of EBM competency. Further work is needed to assess their predictive validity.

## Background

Evidence Based Medicine (EBM) is the triangulation of best available evidence, clinical expertise and patients’ preferences before applying it to clinical decisions [1]. EBM involves five steps: (i) asking the right question; (ii) acquiring evidence; (iii) appraising evidence; (iv) applying to clinical decision and (v) assessing the performance in the first four steps [2]. The importance of ensuring medical students are equipped with the skills to be able to practice EBM has been increasingly recognised in recent years (https://www.gmc-uk.org/-/media/documents/dc11326-outcomes-for-graduates-2018_pdf-75040796.pdf). The General Medical Council (GMC) recommends that ‘Newly qualified doctors must be able to apply scientific method and approaches to medical research and integrate these with a range of sources of information used to make decisions for care’ (https://www.gmc-uk.org/-/media/documents/dc11326-outcomes-for-graduates-2018_pdf-75040796.pdf). It should be noted that the term Evidence Based Medicine is used inter changeably with evidence-based practice (EBP) and evidence-based healthcare (EBHC), but for the purpose of this study we have used the term Evidence Based Medicine.

An evidence-based approach is considered a core competency for clinicians and tremendous efforts have been made to embed EBM training in both undergraduate and postgraduate medical curricula [3, 4]. Various options of teaching EBM in undergraduate medical curriculum have been explored, ranging from standalone courses to those integrated with clinical teaching [4–6]. Teaching EBM as a longitudinal theme across the medical curriculum has been shown to be effective in improving EBM knowledge, as demonstrated by students’ performances in the validated Fresno and Berlin tests [7]. Evidence further supports the view that EBM teaching and learning strategies should focus on implementing multi-faceted, clinically integrated approaches with assessments of knowledge, skills and behaviour in the medium to long term using validated assessment tools [5].

While designing an effective EBM curriculum, in addition to adopting effective teaching methods, medical educators need to ensure assessment of EBM competencies is incorporated into the assessment strategy and blueprinting. Medical educators use a variety of assessments to evaluate EBM competencies of medical students, though knowledge based testing using written assessments has been the traditional method to assess EBM competence [8].

Guidance has already been developed for classification of tools to assess evidence based practice (EBP) learning, which recommend a common taxonomy and propose a framework -CREATE (Classification Rubric for Evidence Based Practice assessment tools in Education) for classifying such tools [9]. The framework has seven categories of EBM learner educational assessments (reaction to educational experience, attitudes, self-efficacy, knowledge, skills, behaviours, and benefits to patients) and the five steps of EBM. Despite the increasing integration of evidence-based practice in healthcare education, there are limited assessment tools with established psychometrics [9]. Current tools are often focussed on the knowledge and skills domains – with a dearth of validated tools that can assess (i) performance of EBP skills and ability to obtain and integrate patients’ values and perspectives in the context of EBP; (ii) monitor learners’ EBP behaviours in high stakes assessments and (iii) measure patient outcomes.

More recently, core competencies were published for health professionals in EBP [10]. The authors have recommended a consensus set of 68 essential core competencies that should be taught in EBP educational programmes. The competencies have been grouped into the main EBM domains and details on the level of delivery of teaching of each competency have been provided- ‘mentioned’, ‘explained’ or ‘practiced with exercises’, meant to serve as a proxy for the time to be spent in teaching each competency. EBP educators are encouraged to map their curricula to these competencies and identify any gaps in coverage of essential content. This set has been recommended as one of several steps in aiding EBM educators move towards a competency based EBP education.

Furthermore, assessment in medical education is evolving; moving from an assessment of knowledge to assessment of performance [11]. Assessment of performance in medical education depends on the choice of appropriate tools to measure relevant educational outcome domains. While written assessments provide an opportunity to demonstrate knowledge at Miller’s assessment of “knows” and “knows how”; objective structured clinical examinations (OSCEs) provide an opportunity to demonstrate skills at Miller’s assessment of “shows how” and “does” in addition to testing core knowledge. OSCEs also provide an opportunity to assess such skills in a simulated environment that is closest to real-life settings that students will encounter in future clinical contexts [12].

OSCEs have been used to evaluate various EBM competencies in medical students such as simple critical appraisal skills [13]; the first three steps of asking, acquiring, and appraising [14]; and asking, acquiring, appraising and applying to simulated clinical scenario [15, 16]. However, studies published to date have often presented a single OSCE station, often limited to assessing only the first few steps of EBM. When EBM is integrated as a longitudinal theme into the undergraduate course, it is helpful to have an EBM assessment regime that examines students’ mastery of EBM competency as they progress through the undergraduate curriculum. EBM educators should develop specific assessment tools that provide accurate, reliable, and timely evaluation of EBP competencies of learners [10].

The aim of this study was to develop and evaluate the feasibility of a suite of EBM OSCEs for use in undergraduate medical education. A spiral model of OSCEs with increasing complexity, has been proposed alongside mapping them against the core competencies for EBP and classified against the CREATE framework. This model can be applied not only to undergraduate medical education but to also to any environment where the use of evidence-based practice is paramount.

## Methods

### EBM curriculum in UBMS

**Fig. 1:**
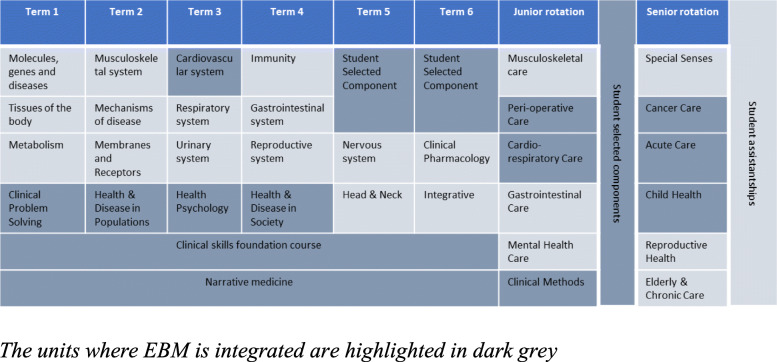
EBM integrated as a longitudinal theme in UBMS

The University of Buckingham Medical School (UBMS) is a relatively new medical school, with the first intake of students in January 2015. The EBM curriculum is based on the learning outcomes from GMC’s Outcome for Graduates, 2018 with the EBM curriculum integrated as a multifaceted, clinically integrated longitudinal theme into the broader undergraduate medical curriculum. The MBChB course at UBMS consists of two phases. Phase I, the first 2 years of the MBChB course, has a focus on biomedical sciences with some patient interactions. Phase II continues for the next two and a half years until graduation during which the students are in clinical placements in hospitals and primary care. EBM has been integrated longitudinally across the MBChB course, beginning in term 1 of Phase I (Fig. 1).

**Fig. 2:**
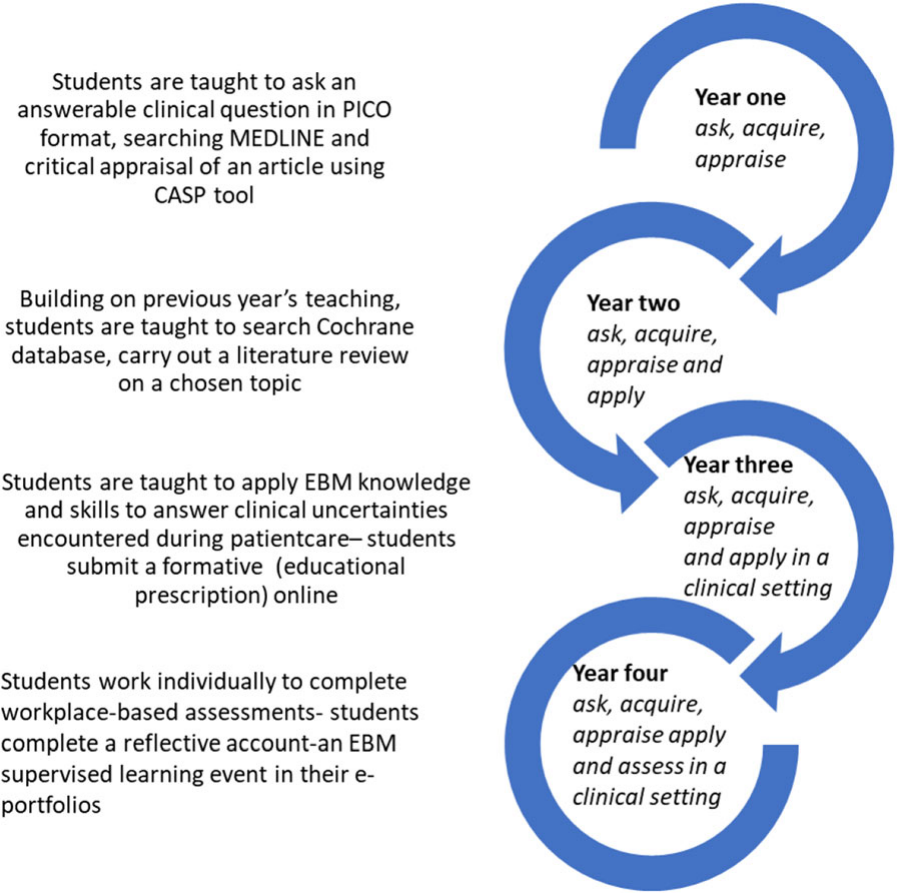
Spiral EBM curriculum in MBChB course in UBMS

Early findings of the evaluation of the curriculum in the initial 2 years have shown it is effective in improving students’ EBM knowledge as measured by change in the students’ performance in Fresno test [17]. Blended teaching methods have been implemented involving the integration of online and face to face teaching activities. Small group tasks are based on clinical vignettes and flipped classroom methods have been introduced to ensure students receive an education tailored to their individual needs. EBM teaching starts in the first term with asking an answerable clinical question, progressing onto literature searching workshops and critical appraisal of scientific articles. Students are taught to apply the findings to simulated scenarios in the first and second year of the curriculum. When students move to clinical rotations in their third and fourth years, they are asked to apply their EBM knowledge and skills in real life clinical scenarios. Details of the curriculum along with the evaluation of the effectiveness using validated tools such as the Fresno been described in a previous publication [17]. A spiral approach to the EBM curriculum has been implemented, where teaching of EBM concepts and applications increase in complexity and are reinforced longitudinally throughout the curriculum (Fig. 2).

Students continue to elaborate on previous topics while building their competency in EBM knowledge and skills. In year one students work in groups to develop answerable clinical questions in PICO format, acquire evidence from MEDLINE, and critically appraise primary articles. Students present findings to the rest of the class, are graded on their formative assignment and receive feedback. In addition, students are introduced to shared decision making in lectures and shown examples of patient decision aids and shared decision-making YouTube videos. In year two, students continue to work in their groups to learn searching for evidence from Cochrane database, critically appraise evidence for a literature review on a topic of clinical uncertainty. Students submit a poster on this literature review, which is graded, and feedback provided. We continue to share examples of shared decision making by using YouTube videos developed by our students in their final years and junior doctors (who are graduates from our school), based on their experiences in clinical placements.

In year three, students identify a clinical uncertainty in their clinical placements, work individually, in pairs or smaller groups to search for evidence, carry out a brief appraisal before applying findings to the clinical uncertainty. This might be before or after the clinical decision is made and students complete an online educational prescription [18]. In the fourth year, students work individually to identify a clinical uncertainty, search for pre-appraised evidence such as Cochrane, NICE, UpToDate to find answers, apply findings to their question and complete an EBM supervised learning event (workplace-based assessment) in their e-portfolio with a reflection on their task.

In UBMS, while establishing the EBM curriculum, EBM focused written assessments and OSCE stations of varying complexity have been integrated into the MBChB course. Psychometric performance of all EBM assessments is routinely analysed.

#### Written assessments

Throughout the course students are required to sit summative written assessments, termly in phase 1 and yearly in phase 2. EBM questions within the summative written examinations are usually short answer questions, single best answer style questions, or ‘fill in the blank’ style questions testing students’ knowledge and application of EBM including simple epidemiological calculations and interpreting statistics. The questions follow the ask, acquire, appraise, and apply of the CREATE framework increasing in complexity and integration with clinical tasks as the student’s progress throughout the course.

#### Objective structured clinical examinations (OSCE)

The annual summative OSCEs at UBMS are blueprinted across the whole curriculum and test students across all taught units and themes, including EBM. EBM-based OSCE stations are created and scripted by the Public Health and EBM theme lead and public health trainees in collaboration with the Assessment Lead. In total, 29 public health and EBM based OSCE stations have been developed and added into our bank of 299 OSCE stations (ie public health and EBM OSCE stations constitute 10% of all OSCE stations). Of the 29 public health and EBM based stations, six stations which were purely EBM based and were used for summative assessments, have been included in this study. Different content experts wrote the OSCE stations which were all piloted before their use in summative OSCEs. The stations were based largely on real life scenarios and any station that contained a new assessment format was piloted before use. Based on feedback from the pilot, changes were made to the content and presentations of stations before final use in summative assessments. Development of the OSCE stations in collaboration with content experts and the associated feedback loops, assured us of face validity. The EBM OSCE stations were integrated into Phase 1 (year 1 and 2) summative 12 station OSCE diets and Phase 2 (clinical) 10 station OSCE diets during both Intermediate Professional examination (third year)  and the Final Professional Examinations (fourth year). As a result, there needed to be 4 levels of complexity to the stations to ensure that we were testing students’ mastery of EBM from novice towards expert.

Content validity of OSCEs can be measured by using feedback from expert opinion [16]. All our OSCE stations were developed by EBM experts and piloted with a small cohort before being used in our high stake’s assessments. We optimised the reliability of OSCE stations by having standardised scoring rubrics and trained assessors and standardised patients. We offered robust examiner training to minimise examiner variation in scoring and ensured consistency in examiner behaviour through calibration and ‘roving moderation’. By using different examiners for our OSCE stations, we minimised individual assessor bias.

During the development of EBM stations, an increasing complexity of the task was developed from focusing more on literature searches in the early years to explaining complex epidemiological terms to standardised patients in later years where the trained standardised patients played the role of patients in simulated hospital and primary care settings. In the early years, the simulated patients were instructed to ask queries regarding new evidence on treatments. In the later years’ stations, students took a focused history, read relevant literature (which were provided) and explained or applied the findings to the patient or the patient’s carer. Students were required to sit summative OSCEs at the end of each year of the MBChB course. Each OSCE station was 8 minutes long in the early years of the course and 10 minutes long in the later years. OSCEs in UBMS are all domain scored against four competencies/domains: (i) communication skills, (ii) clinical knowledge and problem solving, (iii) practical skills, and (iv) professionalism. The examiners directly observed the students and gave a score for each domain ranging from 1 (poor) to 5 (excellent) and an overall global grade against a 6-point scale ranging from very poor to excellent. A full training package was provided for all assessors and simulated patients prior to the OSCEs. In addition, there was an extensive on-the-day calibration exercise where assessors reviewed the station to generate word pictures depicting differing levels of performance (score of 1,3 and 5) in each domain to ensure group think between the assessors and minimise inter assessor variance. Moderation of the stations and assessor performance was undertaken using ‘roving moderators’ who led the calibration exercise and reviewed the stations live across all the concurrent circuits. The cut scores were generated for each station every time it was used using Borderline Regression methodology.

### Ethics

Ethical approval for the study was provided by the University of Buckingham School of Science and Medicine Ethics Committee. All students were invited to participate in the study and were introduced to the study purpose through a verbal presentation at the beginning of phase I EBM teaching. Further details were provided via the virtual learning environment and students had an option to opt out of the study without giving a reason. Informed consent was obtained from participants at the start of the study. All participants data was anonymised before analysing the data and they were assured that only anonymised data would be published.

### Analysis

The station performance data was analysed using the Classical Test Theory, reviewing the mean station domain score, pass rates and item total correlation (ITC) [19]. The ITC was calculated for each OSCE station in relation to the overall student performance across all OSCE stations. Conversely, the Cronbach’s alpha represents the reliability for each individual station. Standard setting was completed using the borderline regression methodology [20]. All analyses were carried out in SPSS v 26 and STATA 16.1. The OSCEs were mapped against the categories in the CREATE framework, based on- their relevance to the five steps of EBM and the seven learner educational assessments. CREATE framework uses the terms- asking, searching, appraising, integrating and evaluating for the five steps of EBM. For simplicity and to be consistent, we have used the terms originally proposed- asking, acquiring, appraising, applying and assessing while mapping our OSCEs to the framework. We then reviewed the EBM competencies tested by these OSCE stations against the EBP core competency framework; proposed a new model for integrating EBM OSCEs of progressive complexity in our assessment strategy in UBMS.

## Results

Details of the six OSCE stations and their corresponding performance data is provided in Table 1 below. There were two stations administered for first year students, two stations for second year students and one each for the third- and fourth-year students. The number of students in each cohort varied from 59 to 81.

**Table 1:** Psychometric test results from the EBM OSCE stations

OSCE Identifier	Year	EBM task	Station summary	Number of students	Cronbach’s alpha	ITC	Mean score (out of a maximum score of 20)	Cut score	Number failing
1	year 1	Asking, acquiring	Students were asked to formulate an appropriate clinical question and search terms. They were asked to search the database PubMed, while explaining what they were doing to the examiner.	63	0.81	0.48	15.71	9.6	4
2	year 1	Appraising	A patient presented for review and asked explanation for some epidemiological terms in an article.	81	0.85	0.494	13.58	12	19
3	Year 2	Appraising	A patient had found some information on the internet and wanted to discuss the article so she could make the right decision.	61	0.74	0.311	13.33	11	13
4	Year 2	Appraising	A patient wanted to discuss his screening results and understand what the findings meant.	71	0.73	0.367	15.61	13	13
5	Year 3	Appraising and applying	The student was assessed on his/her skill to apply the findings in shared decision making with the patient to manage their condition.	64	0.75	0.064	16.83	13	6
6	Year 4	Applying	A patient had found information on the internet and was concerned that the vaccinations her child had had might have caused her child’s delay in talking.	59	0.67	0.205	15.46	11	1

Students’ performance against the four competencies/domains remained consistent, despite varying degrees of complexity of stations across the years. This demonstrated that the stations could progressively assess the students’ developing EBM competencies, alongside assuring us of their performance against the four key competencies. High reliability and consistency were demonstrated for the OSCE circuits (Cronbach’s alpha = 0.67–0.85). Except for one OSCE station, all stations had excellent correlation coefficients and hence a high reliability, ranging from 0.21–0.49. It is thought that the station that did not perform as well was perhaps slightly too easy. The domain mean score ranged from 13.33 to 16.83. The resources needed to run these stations were the same as any standard communication skills OSCE stations, making them feasible.

Having tested the psychometric properties of the OSCE stations, we then mapped our EBM OSCEs against the categories of the CREATE framework. Of the seven assessment categories, our OSCEs assess knowledge, skills, and behaviour. Of the five steps of EBM, our OSCEs can assess four of them- asking, acquiring, appraising, and applying evidence in practice. The classification of our EBM OSCEs against the CREATE framework is shown in Table 2.

**Table 2:** EBM OSCEs in UBMS mapped against the seven educational domains and the five EBM steps (CREATE framework)

Assessment category		Type of assessment	Steps of EBM				
**7**	**Benefits to patients**	Patient -oriented outcomes					
**6**	**Behaviours**	Activity monitoring	✓	✓	✓	✓	
**5**	**Skills**	Performance assessment	✓	✓	✓	✓	
**4**	**Knowledge**	Cognitive testing	✓	✓	✓	✓	
**3**	**Self-efficacy**	Self-report/Opinion					
**2**	**Attitudes**						
**1**	**Reaction to the educational experience**						
			**Ask**	**Acquire**	**Appraise**	**Apply**	**Assess**

Table 3 illustrates a proposed spiral model for assessing EBM using OSCEs. This model uses OSCEs as the assessment tool to progressively assess the five steps of EBM as students move from first year to the final year of their undergraduate curriculum. Whilst many of the EBM skills are assessed in stations across all years of the course, the proposed hierarchy enables assessing increasing complexity, accounting for the learner’s journey from novice to expert. The hierarchy has been plotted against the core competencies for EBP, as illustrated by Albarqouni et al., focusing on the competencies that required to be practiced with exercises, thus applying them to a more complex and spiral structured approach to assess students’ practical skills in EBM.

**Table 3:** Proposed spiral approach of EBM OSCE stations of increasing complexity for different years of undergraduate medical education mapped against the core competencies

Year	EBM task	Mapping against the EBP core competencies (focusing on those rated by authors as ‘practiced with exercises’)	Possible EBM station	The spiral approach to assessment of EBM skills in UBMS
year 1	Asking Acquiring	Convert clinical questions into structured, answerable clinical questions using PICO Construct and carry out an appropriate search strategy	Students formulate an appropriate clinical question and search terms which they apply to a database search	Examine Evidence
Year 2	Appraising	Interpret commonly used measures of uncertainty, in particular, confidence intervals Interpret different types of measures and effect, including key graphical presentations Critically appraise and interpret a treatment study	Students explain epidemiological terms in an article to a patient.	Consider evidence
			Students review clinical guidelines and apply it to the diagnosis and management of a clinical condition.	
Year 3	Appraising Applying in a simulated clinical setting	Interpret different types of measures and effect, including key graphical presentations Critically appraise and interpret a systematic review Critically appraise and interpret a treatment study	Students interpret and explain screening results to a patient using epidemiolocal terms within their explanations Students discuss a paper with a patient to discuss the management of the disease	Utilise evidence as a tool
Year 4	Applying	Engage patients in a decision-making process, using shared decision making, including explaining the evidence and integrating their preferences	Students explain epidemiological terms mentioned in publications to help guide shared decision making with the patient.	Integrate evidence
			Students use a patient decision aid to guide a shared decision-making discussion with a patient.	

## Discussion

Six EBM based OSCE stations assessing various stages of EBM were created for use in an undergraduate medical school, all of which had good psychometric properties. The OSCE stations were classified according to the CREATE framework and mapped against the core competencies for EBP. A spiral model of OSCEs of increasing complexity was proposed to assess EBM competency as students progressed through the MBChB course.

With the use of EBM continuously increasing in clinical practice, medical schools need to ensure tomorrow’s doctors are trained in asking, acquiring, appraising and use evidence in clinical decisions. The importance of EBM is undebatable, and its importance is such that the GMC has cited EBM as an outcome which students must be proficient at prior to graduation (https://www.gmc-uk.org/-/media/documents/dc11326-outcomes-for-graduates-2018_pdf-75040796.pdf). It is well recognised that assessment drives learning [21] and helps steer curricular development through engendering an understanding of students’ grasp of key concepts. Therefore, it is imperative that students are both taught and assessed in EBM skills throughout their medical education.

OSCEs can have a positive impact as they can drive learning [22] and lifelong learning if assessment tasks closely simulate real life practice. There is a need to identify how medical students incorporate EBM skills into clinical practice as they gain greater clinical exposure [12]. Knowledge testing alone does not equate to the ability to apply EBM in real clinical scenarios, medical educators should assess the application of EBM skills in simulated clinical scenarios through OSCEs in addition to assessing knowledge in written tests.

The OSCEs developed in UBMS provided us an opportunity to test students’ EBM skills and behaviour, in addition to knowledge gained. OSCEs also give an opportunity to design stations of increasing complexity and facilitate spiral assessment to map to the spiral curriculum throughout all years of the MBBS course. Previous studies which have shown the feasibility of assessing EBM skills in OSCEs have offered them from the second year of the curriculum [8, 13]. Our study has shown that OSCEs can be introduced from the first year in undergraduate medical education. We have designed our OSCEs to assess students’ developing EBM competencies as they progress through the undergraduate course by identifying and assessing key EBM skills from the novice demonstration of ‘asking’, ‘acquiring’ and ‘appraising’ in early years to emerging professional skills of ‘applying’ of EBM in later years. We have used our summative OSCEs for evaluating students’ EBM competencies and used data from these summative assessments to continuously review the effectiveness of our spiral EBM curriculum. To our knowledge, this is the first time a study has reported the use of a range of EBM OSCE stations in an undergraduate medical school where the OSCEs complement the spiral curriculum of EBM teaching.

In addition to assessing students’ competence in asking, acquiring and appraising evidence, our EBM OSCEs also assessed students’ ability to communicate key statistics such as Relative risk, Odds ratio, 95% Confidence Interval and *p*-values in lay terms to standardised patients, further reinforcing findings from an earlier study that medical educators can assess students’ ability to communicate EBM outcome measures to patients [8]. We are continuing to innovate by exploring other areas of the EBM curricula which could be assessed within OSCE stations such as assessing the students’ ability to use decision aids to enable shared decision making when consulting with patients.

We have demonstrated that it was feasible to assess four EBM competencies (asking, acquiring, appraising, and applying to clinical decisions) using OSCEs of varying complexities throughout the MBChB course. This can be easily transferred to other settings including assessing EBM competencies in postgraduate medical trainees and practicing clinicians. We have also shown the relevance of our EBM OSCEs against the CREATE framework. Of the seven assessment categories, our OSCEs assess knowledge, skills, and behaviour. Of the five steps of EBM, our OSCEs can assess four of them- asking, searching, appraising, and integrating evidence in practice. We hope this helps developers of new EBM assessments to identify and where possible address the current gaps. We have also mapped our EBM OSCE stations against the proposed core competencies in evidence-based practice for health professionals, focusing on those rated by authors as ‘practiced with exercises’. OSCEs and workplace-based assessments are two most used tools available to assess performance in healthcare. In addition to summative EBM OSCEs and written assessments, we have integrated formative assessments such as the Fresno, ACE test and workplace based assessments such as the EPs and EBM supervised learning events (SLEs) in students’ e-portfolios for students to reflect on their experience of applying EBM knowledge and skills in clinical placements. Multiple assessment methods are necessary to capture different aspects of EBM competency. For knowledge and application of knowledge (‘knows’ and ‘knows how’ of Miller’s pyramid), written assessments such as MCQs and short answer questions including the Fresno and ACE are appropriate. OSCEs are ideal assessments for ‘shows how’. Workplace based assessments such as EPs and reflections in e-portfolios are appropriate for assessment of ‘does’ of Millers pyramid. We have built in all the above into our EBM curriculum and are carrying out a programmatic assessment of our curriculum. Each method of assessment provides unique data to inform the decision of whether a candidate has achieved a certain level of competency. Drawing on Kane’s theory of validity, the use of OSCEs, as well as the other mentioned assessment typologies, can only provide a certain level of inference to inform the decision on competency [23]. Further information in ‘real-life’ settings is required in order for a true judgement about the validity of utilising such a programmatic approach to assessing competency in EBM [24].

Public health and Evidence Based Medicine is a longitudinal theme in our school, which enabled integration of EBM into teaching and assessments across our MBChB course. In addition to a longitudinal approach to teaching, there is a systematic representation of EBM in all summative assessments. Hence our students had a firm grounding in EBM principles. Whether the spiral approach of EBM OSCE stations is feasible in existing medical schools, which may not have EBM embedded as a theme, is uncertain, but there is no reason to suggest that the findings could not be extrapolated to any medical school. This spiral assessment approach could also be applied to any other longitudinal theme such as clinical skills and communications skills.

This study had some limitations. Faculty development and lack of trained EBM educators (other than the EBM lead) within the school was a challenge in our study. The EBM OSCEs were designed by the EBM theme lead in collaboration with the Assessment Lead and external EBM experts. Issues relating to faculty development was not unique to our setting, it has been identified as a challenge in the delivery of effective EBM curriculum in other medical schools [25]. However, our study has shown that with a strong commitment from a medical school, close collaborative working and a supportive learning environment it is feasible to integrate EBM into the assessment strategy of a medical school.

With an increasing focus on EBM teaching, there is a need for effective and efficient methods for assessment of EBM knowledge and skills. This study along with our previous studies addresses the gap in current evidence relating to embedding evidence-based medicine into all aspects of medical education [6]. The OSCE stations demonstrated excellent reliability and provided evidence for developing a hierarchy of assessing scaffolded learning and mastery of EBM competency. Further work is needed to assess its predictive validity, which would be valuable at identifying ‘at-risk’ students at an early stage and intervening accordingly.

## Conclusions

The use of the OSCEs is a feasible method of authentically assessing leaner EBM performance and behaviour in a high stakes assessment setting. Use of valid and reliable EBM-based OSCE stations provide evidence for continued development of a hierarchy of assessing scaffolded learning and mastery of EBM competency. Further work is needed to assess their predictive validity.

## Data Availability

The data are available to all interested researchers upon request. Please contact the corresponding author.

## Abbreviations

EBM: Evidence Based Medicine
UBMS: University of Buckingham Medical School
OSCE: Objective Structured Clinical Examination
GMC: General Medical Council
EBP: Evidence Based Practice
EBHC: Evidence Based Healthcare
CREATE: Classification Rubric for Evidence Based Practice assessment tools in Education
ACE: Assessing Competency in Evidence Based Medicine
EP: Educational Prescription
ITC: Item Total Correlation
